# Vaccination Directed against the Human Endogenous Retrovirus-K Envelope Protein Inhibits Tumor Growth in a Murine Model System

**DOI:** 10.1371/journal.pone.0072756

**Published:** 2013-08-30

**Authors:** Benjamin Kraus, Katrin Fischer, Sarah M. Büchner, Winfried S. Wels, Roswitha Löwer, Katja Sliva, Barbara S. Schnierle

**Affiliations:** 1 Paul-Ehrlich-Institut, Langen, Germany; 2 Chemotherapeutisches Forschungsinstitut Georg-Speyer-Haus, Frankfurt, Germany; National Institute of Allergy and Infectious Diseases, United States of America

## Abstract

Human endogenous retrovirus (HERV) genomes are chromosomally integrated in all cells of an individual. They are normally transcriptionally silenced and transmitted only vertically. Enhanced expression of HERV-K accompanied by the emergence of anti-HERV-K-directed immune responses has been observed in tumor patients and HIV-infected individuals. As HERV-K is usually not expressed and immunological tolerance development is unlikely, it is an appropriate target for the development of immunotherapies. We generated a recombinant vaccinia virus (MVA-HKenv) expressing the HERV-K envelope glycoprotein (ENV), based on the modified vaccinia virus Ankara (MVA), and established an animal model to test its vaccination efficacy. Murine renal carcinoma cells (Renca) were genetically altered to express *E. coli* beta-galactosidase (RLZ cells) or the HERV-K ENV gene (RLZ-HKenv cells). Intravenous injection of RLZ-HKenv cells into syngenic BALB/c mice led to the formation of pulmonary metastases, which were detectable by X-gal staining. A single vaccination of tumor-bearing mice with MVA-HKenv drastically reduced the number of pulmonary RLZ-HKenv tumor nodules compared to vaccination with wild-type MVA. Prophylactic vaccination of mice with MVA-HKenv precluded the formation of RLZ-HKenv tumor nodules, whereas wild-type MVA-vaccinated animals succumbed to metastasis. Protection from tumor formation correlated with enhanced HERV-K ENV-specific killing activity of splenocytes. These data demonstrate for the first time that HERV-K ENV is a useful target for vaccine development and might offer new treatment opportunities for diverse types of cancer.

## Introduction

Human endogenous retroviruses (HERVs) are relics of evolutionary ancient viral infection events into the germ line which are now transmitted vertically. These retrovirus genomes are chromosomally integrated in all the cells of an individual and their sequences comprise about 8% of the human genome [1]. HERVs are classified by the single letter amino acid code for the tRNA specific for the primer-binding site used to initiate reverse transcription. At present, 11 distantly related HERV groups with a tRNA lysine (K) primer binding site are known (reviewed in [2]). One of these, the HERV-K/HML-2(hom) group, is the only known endogenous retrovirus group encoding all structural and enzymatic proteins. For reasons of simplicity, HERV-K/HML-2(hom) will hereafter be abridged to HERV-K.

Retroviruses have the ability to integrate their sequences into the cellular genome which may result in cell transformation and cancer caused by insertional mutagenesis. Therefore, endogenous retroviruses had to be highly regulated shortly after they entered the human species [3], [4]. Consequently, HERV-K gene expression is generally found to be repressed and immunological tolerance is unlikely. However, HERV-K expression occurs and has been described in conjunction with disease. Reactivation of HERV-K proviruses coding for all viral proteins is well established for human teratocarcinomas [5], melanomas [6], [7] and ovarian cancer [8], [9]. HERV-K ENV has been found to be overexpressed in breast cancer tissues [10], [11]. The overexpression of Gag has been seen in the peripheral blood cells of leukemia patients [12] and also in prostate cancer and ovarian cancer but not in healthy donors [13], [14]. Furthermore, it has been reported that HIV infection leads to increased expression of HERVs, and T cell responses to HERV peptides have been detected in HIV-1-infected patients [15], [16], [17], [18], [19], [20]. Individuals who control HIV-1 viremia without highly active antiretroviral therapy (HAART) had stronger and broader HERV-specific T cell responses than HAART-suppressed patients, virologic noncontrollers, immunologic progressors, and uninfected controls. An inverse correlation between anti-HERV-K T cell responses and HIV-1 viral load has also been detected [21], [22]. Recently, a HERV-K-specific CD8+ T cell clone could be isolated, which eliminated cells infected with a panel of globally diverse HIV-1, HIV-2, and SIV isolates *in vitro* [23]. This indicates a potential therapeutic value of HERV-K-directed T cell responses for HIV-infected patients on the one hand and, on the other, suggests a lack of immune tolerance to HERVs in HIV-infected individuals and most likely also in healthy individuals. High HERV-K-directed T cell responses were not detrimental for the HIV patients and give first indications that a HERV-K vaccine might be safe. Additionally, expression of HERV-K proteins was recently assessed by immunohistochemical analysis of human tissue. Several human tissues expressing HERV-K Gag were identified; however, no healthy human tissue showed HERV-K ENV expression [24]. Another study found HERV-K ENV protein expression in villous and extravillous cytotrophoblast cells of the human placenta [25]. Simian endogenous retrovirus K (SERV-K) expression has been observed in several tissues of nonhuman primates [24]. Vaccination of rhesus macaques which carry SERV-K with SERV-K Gag or ENV induced T cell responses without vaccine-related pathogenicity [24]. In summary, these data support the notion that HERV-K ENV-directed immune responses might be induced safely in humans.

There is good evidence that tumor-associated antigens (TAAs) can be used for therapeutic vaccinations, in particular for the treatment of minimal residual disease. Furthermore, induction of immunological memory may prevent disease relapse. Poxviruses are attractive vaccine vectors. They activate robust cellular MHC class I- and II-restricted CD8+ and CD4+ T cell responses against recombinant antigens. This, for instance, has been shown for NY-ESO-1, a marker for a range of human malignancies [26]. Recombinant vaccinia-NY-ESO-1 and recombinant fowlpox-NY-ESO-1 were used as vaccines in 36 patients with different tumor types. In several patients with melanoma, the natural course of the disease was favorably influenced by vaccination [26]. Likewise, the attenuated modified vaccinia virus Ankara (MVA) has been extensively used as vector for the delivery of TAAs in preclinical studies and clinical trials for the treatment and/or prevention of different cancer types [27]. Due to the excellent safety profile of MVA, also in immunosuppressed patients, along with its retained high immunogenicity, it is widely considered as the vaccinia virus strain of choice for clinical investigation.

Here we generated a recombinant MVA expressing the HERV-K envelope glycoprotein, and tested its ability to act as a cancer vaccine in a syngenic mouse tumor model using HERV-K ENV-expressing murine renal carcinoma cells (Renca).

## Materials and Methods

### Ethics Statement

This study was carried out in strict accordance with the recommendations in the German Animal Licence regulations (Tierschutzgesetz). The protocol was approved by the Committee on the Ethics of Animal Experiments of the Regional Council Darmstadt (Permit Number: V54 19c 20/15 F107/93). All efforts were made to minimize suffering.

### Generation of Recombinant MVA

The HERV-K envelope gene sequences were excised from the plasmid pHKges, containing the HERV-K genome controlled by a CMV promoter, by *Bci*VI*/Sph*I digestion, treated with T4 DNA polymerase to generate blunt ends, and cloned into the blunted *Asc*I site of MVA expression plasmid pVIdHR-P7.5 to generate the MVA vector plasmid pVI-HKEnv. Upon transfection into MVA-infected cells, the plasmid directs insertion of foreign genes into the site of deletion VI within the MVA genome and allows transcription of the HERV-K ENV gene under control of the vaccinia virus-specific promoter P7.5. The recombinant virus MVA-HKenv was generated in BHK-21 cells by transfection with 1 µg of plasmid DNA, infection at an MOI of 0.05 with MVA-IInew isolate, and plaque selection on RK13 cells [28], [29]. BHK-21 and RK13 cells were obtained from American Type Culture Collection (ATCC) (ATCC-CCL-10 and ATCC-CCL-37 respectively). The recombinant MVA genomes were analyzed by PCR to verify HERV-K ENV gene insertion and genetic stability. Multiple-step growth analysis in chicken embryo fibroblasts (CEFs) demonstrated that the replication capacity of MVA-HKenv was slightly less efficient compared to wild-type MVA. MVA preparations for immunizations were generated by amplification in CEFs. MVA and MVA-HKenv were purified by ultracentrifugation through sucrose, reconstituted in 1 mmol/L Tris/HCl (pH 9.0) and quality controlled once more by PCR and for microbial contaminations.

### Western Blot

Western blot was performed with a BIO-Rad semi-dry blotter. Proteins separated by SDS-PAGE were blotted onto PVDF membranes with 50 mM sodium borate pH 9.0, 20% methanol and 0.1% SDS at 100 mA per membrane for 75 min. Afterwards, membranes were blocked with Roti-Block™ and proteins were detected with α-HERV-K ENV monoclonal antibody (HERM-1811-5; Austral biological, Binzwangen, Germany) and the ECL detection system (Amersham, Freiburg).

### Immunofluorescence Staining of Cells

Cells were fixed with 2% paraformaldehyd and HERV-K ENV was detected after successive incubations with an anti-HERV-K ENV antibody (HERM-1811-5) and a TRITC-coupled goat anti-mouse IgG antibody (DAKO, Hamburg, Germany). Additionally cells were stained with DAPI. Cells were examined with a 400×magnification.

### Cell Culture

HEK 293 T (ATCC: CRL11268), BHK21 (ATCC: CCL-10) and RK-13 (ATCC: CCL-37) cells were cultured in complete Dulbecco’s modified Eagle’s medium (DMEM) containing 10% fetal bovine serum and L-glutamine (2 mM). Renca-lacZ (RLZ) cells were cultured in RPMI medium complemented with 10% fetal bovine serum, L-glutamine (2 mM) and 250 µg/ml Zeocin (Invitrogen, Darmstadt, Germany). For the generation of the RLZ-HKenv cell line, MLV-based retroviral vectors were produced encoding a codon-optimized HERV-K ENV gene (provided by R. Löwer) via the pBabe-puro vector. For selection of transduced cells, 1 µg/ml puromycin was added to the RLZ-HKenv medium. The cell lines were propagated applying standard techniques.

### Detection of MVA-specific T Cells

PE-labeled MHC class I pentamers detecting B8R-specific T cells (TSYKFESV-K^b^) in C57BL/6 mice were purchased from ProImmune and used as recommended by the manufacturer using only half of the suggested amount of pentamer.

### Animal Experiments

Female specific pathogen-free 6–8-week-old BALB/c or C57BL/6 mice were purchased from Harland. For tumor transplantations, RLZ-HKenv cells were grown to 60–80% confluence and detached from the culture dish via trypsin digestion. Cells were washed three times with PBS and resuspended in PBS at a density of 1×10^5^ cells/100 µl for therapeutic vaccination experiments, and 5×10^5^ cells/100 µl for prophylactic vaccination experiments. Indicated cell numbers were injected in the tail vein of mice using standard techniques. Mice were immunized with the indicated amounts of recombinant MVA intra-muscularly into the quadriceps muscles of the hind limbs and then weighed every 3 days. Mice were sacrificed at the indicated time points, and the lungs were prepared and incubated in fixing solution (0.2% glutaraldehyde, 2% formaldehyde in PBS) overnight at 4°C. For X-Gal staining, lungs were incubated in staining solution (5 mM potassium ferrocyanide, 1 mM MgCl_2_, 1 mg/ml X-Gal in PBS) for 24 h. Stained lungs were stored in PBS containing 4% formaldehyde.

### T Cell Assay

The specific cytolytic activity of restimulated spleen cells was measured by a flow cytometry-based assay. RLZ-HKenv cells were stained with the cell membrane-labeling dye PKH67 (Sigma-Aldrich, Munich, Germany), following the manufactures’ instructions, and then seeded at a density of 1.25×10^5^ cells per 24 well one day before the assay. Spleens of vaccinated mice were excised and suspended by passing through a 70 µm filter. For the restimulation, 5×10^6^ spleen cells were cocultured with γ-irradiated (100 Gray) RLZ-HKenv cells in the presence of 200 U/ml murine-IFN-γ and 10 U/ml murine IL-2 in 6 wells. After 48 h, splenic cells were seeded on freshly plated γ-irradiated RLZ-HKenv cells. Incubation lasted 4 days in total. The restimulated spleen cells were counted and cocultivated with labeled target cells (RLZ-HKenv or RLZ) at the indicated ratios for 6 h. Subsequently, cells were harvested with trypsin, washed, stained with PI and analyzed directly by flow cytometry, using an LSRII instrument (BD Biosciences, Heidelberg, Germany) and the FACS Diva Software. Target cells were identified by PKH67 fluorescence and killed/lysed target cells were measured as PKH 67- and PI-positive cells.

### Statistical Analysis

All statistical analyses were performed using Prism GraphPad Software (San Diego, CA). Wilcoxon’s *t*-test was used for statistical analyses. In general, means were used and statistical deviations are presented as standard deviation unless otherwise noted. A p-value<0.05 was deemed statistically significant.

## Results

### Construction and Characterization of a Recombinant MVA Expressing the HERV-K Envelope Glycoprotein

**Figure 1:**
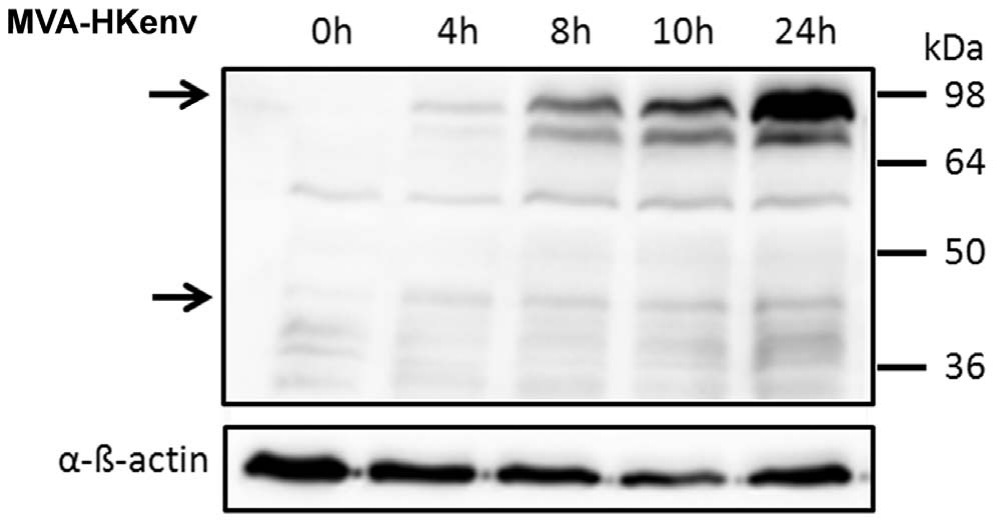
Western Blot analysis of MVA-HKenv-infected 293 T cells. 293 T cells were infected at an MOI of 5 with MVA-HKenv and cell lysates were prepared at the indicated time points. HERV-K ENV was identified with the HERV-K ENV monoclonal antibody HERM-1855 followed by chemiluminescent detection. Arrows indicate the position of HERV-K ENV subunits. Detection of ß-actin was used as loading control.

Like all retroviral envelope proteins, the HERV-K ENV is translated as a precursor protein from a singly spliced transcript. The precursor, with a molecular mass of 90 kDa, is processed into a transmembrane (TM) subunit of 40 kDa with a cytoplasmic and extracellular domain, and a non-covalently linked surface subunit that putatively binds the cellular receptor [30]. The entire open reading frame, which also includes the first exon of the nucleocytoplasmic shuttle protein Rec, was inserted into the MVA genome by homologous recombination using the region flanking deletion VI in the MVA genome. HERV-K ENV gene expression is thereby under the control of the early/late p7.5 promoter [31]. The recombinant MVA (MVA-HKenv) was isolated by K1L selection as described before [29], and HERV-K ENV expression of the final working stock was verified by Western blot analysis of 293 T cells infected at an MOI of 5 (Fig. 1). ENV expression started at 4 h post infection and the protein is partially cleaved, because the precursor as well as the TM subunits were detectable (Fig. 1, indicated by arrows). Two vaccinations on days 0 and 21 of BALB/c mice with 10^7^ IU MVA-HKenv resulted in the weak generation of ENV-specific antibodies by the mice, as analyzed on day 47 by immunofluorescence studies with GH cells, which express high levels of HERV-K ENV protein and are a very sensitive method to detect HERV-K specific antibodies (data not shown) [32]. In addition, nucleolar staining was detectable with one mouse serum, demonstrating an immune response against the co-expressed Rec protein, which is located in nucleoli (data not shown) [33].

### MVA-HKenv Induces Cellular Immune Responses

**Figure 2:**
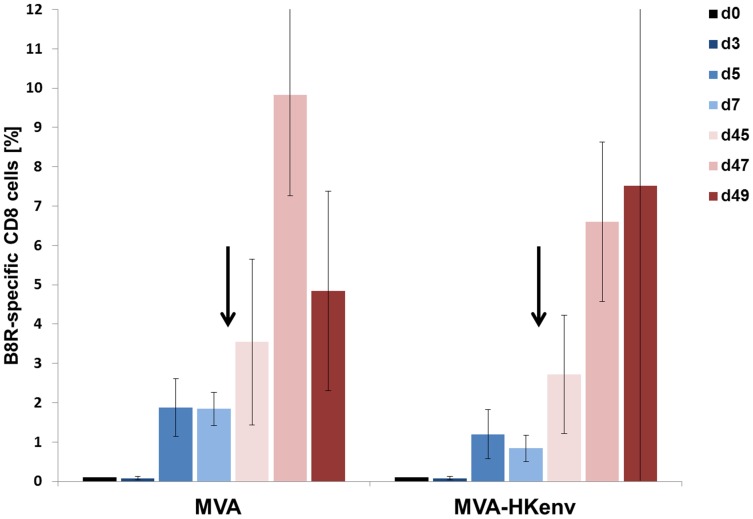
Analysis of B8R-specific CD8+ cells. C57BL/6 mice (n = 5) were injected i.v. with 10^6^ IU MVA or MVA-HKenv. Induction of CD8+ T cells directed against the vaccinia virus B8R gene product was measured at the indicated time points in blood samples by flow cytometry using an MHC class I pentamer. Mice were vaccinated on day 0 and arrows indicate the time point of the second virus application on day 42. The p-values between the two immunization groups were: day 5 p = 0.159; day 47 p = 0.058; day 49 p = 0.472, indicating no significant difference.

Retroviral envelope proteins have been described to have immunosuppressive properties, which have been attributed to interference with natural killer cell and/or CD8+ T cell effector functions [34], [35], [36]. Accordingly, we performed a sequence alignment of the immunosuppressive syncytin-1 sequence (ISD) with HERV-K ENV; however, we could not identify motifs in HERV-K ENV homologous to an ISD sequence. Nevertheless, we examined whether HERV-K ENV expression by MVA interferes with the induction of cell-mediated immune responses directed against MVA. C57BL/6 mice were i.v. infected twice with 1×10^6^ IU MVA or MVA-HKenv, and the induction of MVA-specific CD8+ T cells was monitored by FACS analysis using a TSYKFESV-K^b^ pentamer specific for the immunodominant vaccinia virus B8R-derived T cell epitope. C57BL/6 mice were used, because the vaccinia virus specific pentamer is only commercially available for these mice. B8R-specific CD8+ T cells expanded with similar kinetics after MVA or MVA-HKenv immunization (Fig. 2). The boost immunization on day 42 resulted in a rapid expansion of B8R-specific CD8+ T cells in both groups (Fig. 2, days 45, 47 and 49). The slightly lower values in MVA-HKenv immunized mice were not statistically significant (Fig. 2). Therefore, HERV-K envelope protein expression did not interfere with the development of an adaptive cell-mediated immune response against vaccinia virus and implies that HERV-K-directed CD8+ cells are also likely to be induced.

### Establishment of an Animal Model

**Figure 3:**
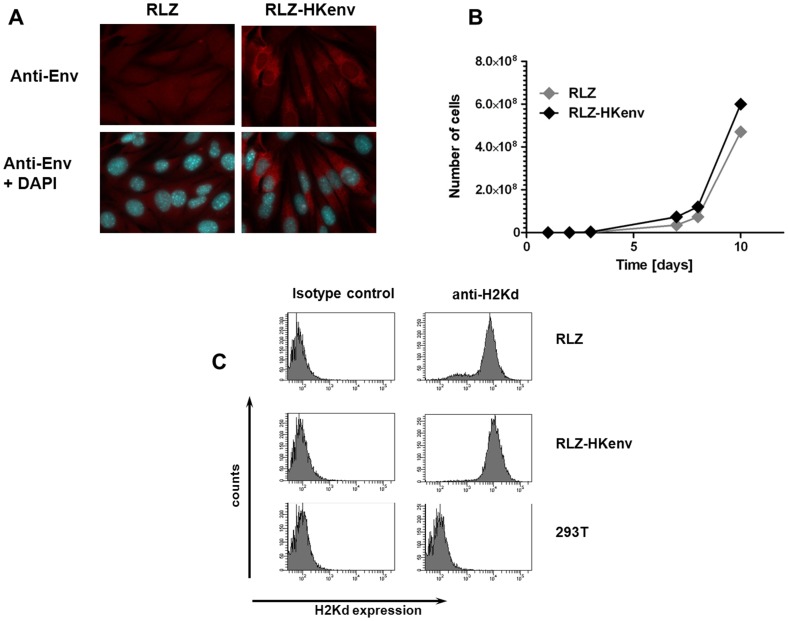
Characterization of RLZ-HKenv cells. A: Immunofluorescence analysis of RLZ cellsCells were fixed and stained either with an anti-HERV-K ENV antibody alone or in combination with DAPI. B: Growth rate of RLZ cells RLZ and RLZ-HKenv cells were counted for 10 days and the number of cells was blotted against the time and gives the growth rate of the cell lines. C: MHC class I expression. MHC class I (H2Kd) expression was analyzed by flow cytometry either with an antibody directed against H2Kd or a control antibody of the same isotype. Human 293 T cells were used as negative control.

A major bottleneck in HERV-K-specific vaccine development is an appropriate animal model, since HERV-K is exclusively expressed in humans. We therefore adapted a syngeneic mouse model for this purpose. Murine renal carcinoma cells (Renca) were genetically altered to express *E. coli* beta-galactosidase (RLZ cells) [37] and the HERV-K ENV gene was then introduced by retroviral transduction (RLZ-HKenv cells). The engineered cell line RLZ-HKenv is positive for ENV expression at various intensities as shown by immunofluorescence analysis and showed a dominant ER/Golgi staining as expected for proteins targeted to the secretory pathway (Fig. 3A). The cells can be passaged without silencing of ENV expression *in vitro* and *in vivo* (data not shown). The growth rate of both cell lines (RLZ and RLZ-HKenv) in tissue culture was comparable and they both expressed MHC class I at similar levels (Fig. 3B and C). Intravenous application of RLZ-HKenv cells into syngeneic BALB/c mice resulted in the formation of pulmonary metastases, which were detectable by X-gal staining upon excision of the lungs.

### Therapeutic Vaccination with MVA-HKenv Reduces the Number of Lung Metastases

**Figure 4:**
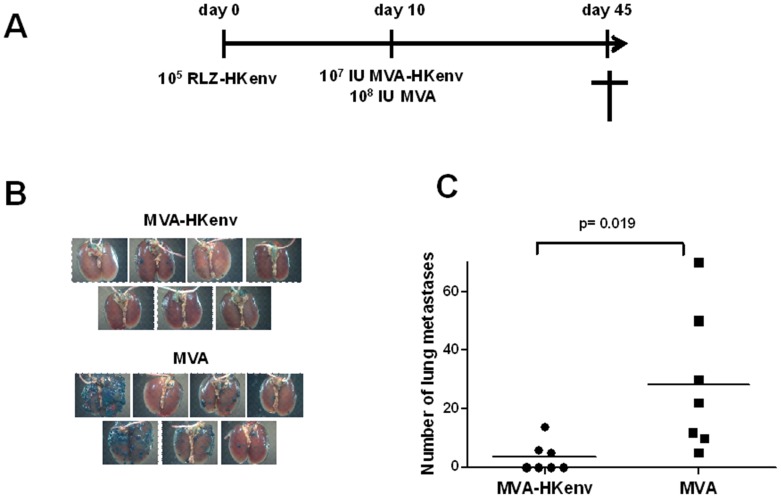
Specificity of the therapeutic vaccination. **A**: Schematic diagram of the therapeutic vaccination Two groups of BALB/c mice (n = 7) were injected i.v. with 10^5^ RLZ-HKenv cells and immunized with either 10^7 ^IU/mouse MVA-HKenv or 10^8 ^IU/mouse MVA i.m. on day 10. The animals were sacrificed on day 45, lungs were removed, and stained with X-Gal. B: Lungs of mice. The lungs of the animals were excised and stained with X-gal. Tumor nodules are visible by blue staining of the cells. C: Analysis of tumor nodulesThe number of tumor nodules was counted on the surface of the lungs. Data for each mouse are shown. The p-value of 0.019 shows significance.

In a therapeutic setting, the vaccination would be used for the treatment of established tumors. Therefore, 10^5^ RLZ-HKenv cells were applied intravenously into BALB/c mice on day 0. Ten days later, after the cells had had the chance to colonize the lung, the mice were vaccinated intramuscularly with either MVA-HKenv or MVA (10^7 ^IU/mouse or 10^8 ^IU/mouse, respectively; n = 7) (Fig. 4A). On day 45, the mice were sacrificed and the lungs were excised, fixed and stained for beta-galactosidase. Only a few pulmonary tumors were detectable in the lungs of MVA-HKenv-vaccinated animals (Fig. 4B). However, MVA-vaccinated animals had a much higher tumor burden. Figure 4C summarizes the number of pulmonary tumors and shows that there was a statistically significant difference in the number of metastases with a p-value of 0.019. A single vaccination with MVA-HKenv dramatically decreased the outgrowth of pulmonary tumors. Still, the number of metastasis showed high variations and therefore we repeated the experiment and observed a similar significant difference. In this experiment, mice treated twice with MVA-HKenv and showed less metastasis compared to MVA treated animals. High numbers of metastasis were only observed in mice treated with MVA.

### Prophylactic Vaccination with MVA-HKenv Prevents the Establishment of HERV-K-Expressing Tumor Metastasis

**Figure 5:**
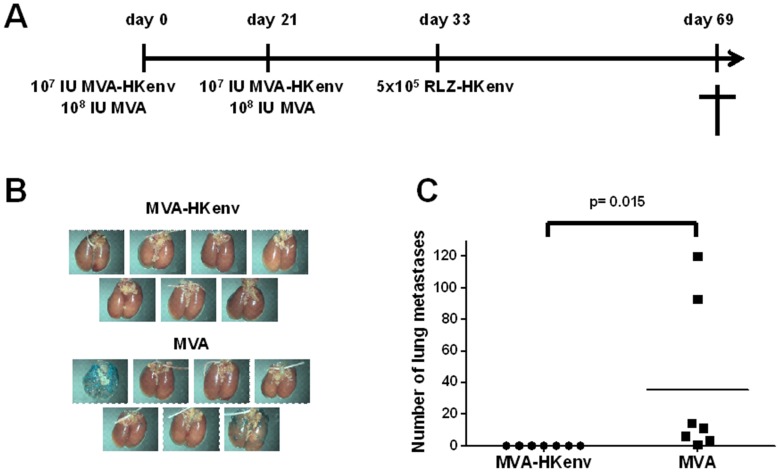
Specificity of the prophylactic vaccination. A: Schematic diagram of the prophylactic vaccination. Two groups of BALB/c mice (n = 7) were immunized i.m. with either 10^7 ^IU/mouse MVA-HKenv or 10^8^ IU/mouse MVA on day 0 and day 21. The mice were injected i.v. with 5×10^5^ RLZ-HKenv cells on day 33 and sacrificed on day 69. The lungs were removed and stained with X-Gal. B: Lungs of miceThe lungs of the animals were excised and stained with X-gal. Tumor nodules are visible by blue staining of the cells. C: Analysis of tumor nodules. The number of lung tumor nodules was counted on the surface of the lungs and data for each mouse are shown. The p-value of 0.015 shows significance.

Cancer vaccination might also be envisioned as a prophylactic treatment of healthy persons with an elevated genetic risk of cancer development. To test the prophylactic properties of MVA-HKenv, BALB/c mice (n = 7) were injected intramuscularly on day 0 with 10^7^ IU MVA-HKenv or 10^8^ IU MVA and boosted with the same dose on day 21. On day 33, the mice were injected i.v. with 5×10^5^ RLZ-HKenv cells and finally sacrificed on day 69. One mouse died on day 68 in the MVA-vaccinated group. Figure 5A summarizes the vaccination schema. Vaccination with MVA-HKenv completely prevented the formation of RLZ-HKenv lung metastasis, whereas MVA-vaccinated animals had a high tumor burden in their lungs (Fig. 5B). The difference between MVA and MVA-HKenv vaccination was statistically significant with a p-value of 0.015 (Fig. 5C) and clearly shows the capability of the vaccination to prevent the establishment of lung metastasis in this aggressive mouse model. Similar results were obtained in a second experiment with a challenge dose of 1×10^6^ RLZ-HKenv cells. Again the MVA-HKenv-vaccinated group did not support the colonization of RLZ-HKenv cells, whereas the MVA-vaccinated group had an average of 21 lung surface metastases.

### MVA-HKenv Vaccination Induces Cytotoxic Lymphocytes able to Kill RLZ-HKenv Cells

**Figure 6:**
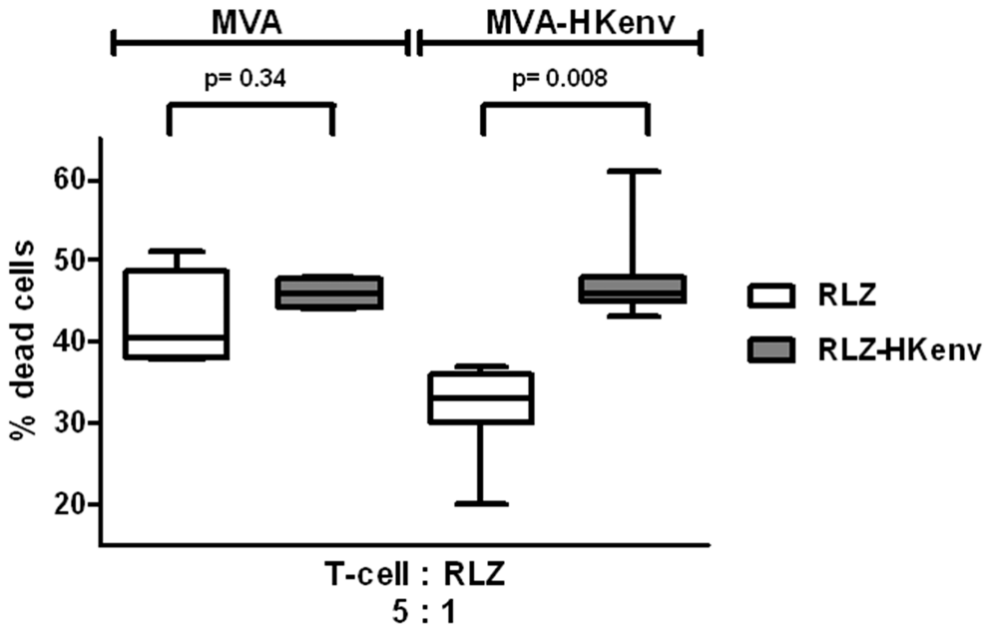
Induction of HERV-K ENV-specific cytotoxic activity in immunized mice. Spleen cells were isolated at the end of the experiment described in Figure 4. Cells were restimulated and their specific cytolytic activity against RLZ-HKenv cells was measured by flow cytometry. RLZ-HKenv and RLZ cells were stained with the cell membrane-labeling dye PKH 67 and restimulated spleen cells were cocultivated with labeled RLZ-HKenv/RLZ target cells at a 5∶1 ratio for 6 h. Subsequently cells were stained with propidium iodide (PI) and directly analyzed by flow cytometry. Dead RLZ-HKenv cells are PKH 67- and PI-positive and the mean values with standard deviations for each group are indicated. MVA-vaccinated group p = 0.34; MVA-HKenv-vaccinated group p = 0.008.

To shed light on the underlying mechanism of tumor rejection, cellular immune responses were analyzed with splenocytes of animals that had undergone prophylactic vaccination. First, cytolytic effector cells were restimulated by co-cultivation of spleen cells isolated on day 69 from prophylactically vaccinated animals with γ-irradiated RLZ-HKenv cells and proinflammatory cytokines for 4 days. The restimulated effector cells were harvested on day 4. After removal of dead cells, they were cocultured at an effector to target ratio of 5∶1 with PKH 67-labeled RLZ-HKenv or RLZ cells for 6 h. The number of dead target cells was determined by flow cytometry as the number of propidium iodide (PI) - and PKH 67-stained double positive cells. Vaccination with MVA-HKenv induced a higher specific cell killing of RLZ-HKenv cells compared to RLZ cells (Fig. 6). In contrast, MVA-vaccinated animals mounted similar cell mediated cytotoxic activities but towards both cell lines, RLZ and RLZ-HKenv, and no difference in killing activity between RLZ-HKenv and RLZ cells could be observed (Fig. 6). Since these mice had a constant exposure to RLZ cells due to the high tumor burden during the experiment and at the time point of lymphocyte isolation, it is likely that tumor growth generated an unspecific immune response towards RLZ cells, which, however, was not early or strong enough to control tumor cell growth. However, exposure of the mice to a specific vaccine prevented tumor outgrowth and induced specific cell-mediated cytotoxic effector functions against HERV-K ENV-expressing tumor cells.

## Discussion

Cancer immunotherapy relies on the tight association of the expression of the target gene in the diseased tissue with its lack in healthy somatic cells. The envelope protein of the human endogenous retrovirus K fulfills this criterion perfectly. Support comes from recently published studies which describe that HERV-K ENV expression is virtually absent in healthy human tissue [24], [20], however care should be taken to exclude persons with other HERV-K expression associated diseases as for instance auto immune diseases [38]. Although another publication described HERV-K ENV expression in human placenta [25], the above authors could not confirm these data [24]. Yet, HERV-K ENV expression is connected with disease and has been observed in diverse types of human tumors and, in addition, in HIV-1-infected patients. However, the exact mechanism of HERV-K reactivation and its functional consequences are still unknown. Currently, HERV-K reactivation is assumed to be a result of deregulated epigenetic silencing mechanisms which still need to be studied in more detail.

Besides tumor-specific protein expression, immune responses to HERV-K have been found in tumor patients. For instance, anti-HERV-K antibodies and T cell responses have been detected in patients with breast and ovarian cancer but not in healthy female controls [8], [10]. HERV-K seropositivity is both a diagnostic and prognostic marker for germ cell tumors (GCTs). There is a strong association of HERV-K antibodies with the clinical manifestation of the disease as well as therapy failure, and significant seronegativity for healthy donors [39]. The negative correlation indicates that HERV-K-directed immune responses have to be elicited early during tumor formation and with high potency to be able to compete with tumor growth. Analyzing HERV-K seropositivity in melanoma patients has revealed an epitope suitable as a melanoma marker [40]. In addition, a humoral immune response against HERV-K gene products significantly correlated with a reduced survival probability of melanoma patients [7].

Another pathological state associated with HERV-K expression is HIV-1 infection, where multiple reports suggest that endogenous retroviruses might be a useful and invariable target for immunotherapy [41]. In this case, cell-mediated immune responses directed against HERV-K correlated with control of HIV-1 viremia [21], [22].

At least 91 full-length HERV-K elements have been reported to exist in the human genome [1] however they accumulated mutations to render them non-infectious. In addition about 2500 HERV-K solitary LTRs exist in the human genome, indicating that they originated from recombinations of full-length HERV-K genomes. This complex nature of HERV-K should be considered for vaccine development and might indicate that for tumor vaccination the here described vaccine based on HERV-K 108 is the best choice. Recently, the activation of a novel HERV-K provirus, K111, was found to be activated by HIV infection and might require using the K111 ENV for vaccine purposes targeting HIV-infected cells [42].

Here, we generated a HERV-K ENV-directed vector vaccine based on the modified vaccinia virus Ankara (MVA), a highly attenuated vaccinia virus strain suitable for clinical application. MVA does not replicate in human cells but shows high protein expression and, consequently, has a very good safety profile without compromising vaccination efficiency. Due to the attenuation, MVA can be used for vaccinations of immunosuppressed patients [43], [44]. Vaccine efficacy testing of the recombinant MVA-HKenv was performed in a surrogate mouse model, using a syngeneic mouse tumor cell line which was genetically engineered to express HERV-K ENV at different protein levels. Although this model is quite artificial, it may well reflect the situation in humans. Since HERV-K expression is normally repressed, it is expected that humans are not tolerant to HERV-K gene products, and immune responses are readily seen in cancer and HIV-infected patients. Formation of pulmonary metastasis in the mice was used as a measure for vaccine efficiency. In a therapeutic setting, after the colonization of RLZ-HKenv cells, a single vaccination of the animals with MVA-HKenv significantly diminished the number of metastases compared to MVA-vaccinated animals. In addition, the prophylactic vaccination, this time with two vaccine applications, completely abolished the formation of lung metastases in the MVA-HKenv-vaccinated group, whereas the MVA-treated group succumbed to tumors. The MVA-HKenv-treated animals developed cell-mediated cytolytic activity against RLZ-HKenv cells, which is most likely responsible for tumor rejection. The experimental vaccine MVA-HKenv may therefore represent a novel treatment for HERV-K-positive tumors or even the HIV-1 infection.

The challenging next step is now to transfer this vaccination strategy to a clinical situation. Side effects of an HERV-K-specific vaccination can only be studied in humans. Primates encode very similar endogenous retroviral genomes and could be used as a model for safety studies [45], [46], [47], [24]. We showed proof of principle for an HERV-K cancer vaccine in a mouse tumor model. In addition, this treatment might also be applicable as an anti-HIV vaccine, the development of which is still hampered by the rapid escape of HIV from antiviral host responses. HERV-K ENV would be available as a constant target for HIV vaccine development, and can expected to be effective, since HERV-K-directed cytotoxic T cell responses have been shown to correlate with mild disease progression in AIDS patients [21].

## References

[1] Subramanian RP, Wildschutte JH, Russo C, Coffin JM (2011) Identification, characterization, and comparative genomic distribution of the HERV-K (HML-2) group of human endogenous retroviruses. Retrovirology 8: 90. 22067224 10.1186/1742-4690-8-90 PMC3228705

[2] Bannert N, Kurth R (2006) The evolutionary dynamics of human endogenous retroviral families. Annu Rev Genomics Hum Genet 7: 149–173. 16722807 10.1146/annurev.genom.7.080505.115700

[3] Rowe HM, Trono D (2011) Dynamic control of endogenous retroviruses during development. Virology 411: 273–287. 21251689 10.1016/j.virol.2010.12.007

[4] Jha AR, Nixon DF, Rosenberg MG, Martin JN, Deeks SG, et al (2011) Human endogenous retrovirus K106 (HERV-K106) was infectious after the emergence of anatomically modern humans. PLoS ONE 6: e20234. 21633511 10.1371/journal.pone.0020234 PMC3102101

[5] Lower R, Lower J, Frank H, Harzmann R, Kurth R (1984) Human teratocarcinomas cultured in vitro produce unique retrovirus-like viruses. J Gen Virol 65 (Pt 5): 887–898. 10.1099/0022-1317-65-5-887 6202829

[6] Buscher K, Trefzer U, Hofmann M, Sterry W, Kurth R, et al (2005) Expression of human endogenous retrovirus K in melanomas and melanoma cell lines. Cancer Res 65: 4172–4180. 15899808 10.1158/0008-5472.CAN-04-2983

[7] Hahn S, Ugurel S, Hanschmann KM, Strobel H, Tondera C, et al (2008) Serological response to human endogenous retrovirus K in melanoma patients correlates with survival probability. AIDS Res Hum Retroviruses 24: 717–723. 18462078 10.1089/aid.2007.0286

[8] Wang-Johanning F, Liu J, Rycaj K, Huang M, Tsai K, et al (2007) Expression of multiple human endogenous retrovirus surface envelope proteins in ovarian cancer. Int J Cancer 120: 81–90. 17013901 10.1002/ijc.22256

[9] Herbst H, Kuhler-Obbarius C, Lauke H, Sauter M, Mueller-Lantzsch N, et al (1999) Human endogenous retrovirus (HERV)-K transcripts in gonadoblastomas and gonadoblastoma-derived germ cell tumours. Virchows Arch 434: 11–15. 10071229 10.1007/s004280050298

[10] Wang-Johanning F, Frost AR, Jian B, Epp L, Lu DW, et al (2003) Quantitation of HERV-K env gene expression and splicing in human breast cancer. Oncogene 22: 1528–1535. 12629516 10.1038/sj.onc.1206241

[11] Ejthadi HD, Martin JH, Junying J, Roden DA, Lahiri M, et al (2005) A novel multiplex RT-PCR system detects human endogenous retrovirus-K in breast cancer. Arch Virol 150: 177–184. 15449135 10.1007/s00705-004-0378-8

[12] Depil S, Roche C, Dussart P, Prin L (2002) Expression of a human endogenous retrovirus, HERV-K, in the blood cells of leukemia patients. Leukemia 16: 254–259. 11840292 10.1038/sj.leu.2402355

[13] Ishida T, Obata Y, Ohara N, Matsushita H, Sato S, et al (2008) Identification of the HERV-K gag antigen in prostate cancer by SEREX using autologous patient serum and its immunogenicity. Cancer Immun 8: 15. 19006261 PMC2935773

[14] Goering W, Ribarska T, Schulz WA (2011) Selective changes of retroelement expression in human prostate cancer. Carcinogenesis 32: 1484–1492. 21828060 10.1093/carcin/bgr181

[15] Contreras-Galindo R, Lopez P, Velez R, Yamamura Y (2007) HIV-1 infection increases the expression of human endogenous retroviruses type K (HERV-K) in vitro. AIDS Res Hum Retroviruses 23: 116–122. 17263641 10.1089/aid.2006.0117

[16] Contreras-Galindo R, Kaplan MH, Markovitz DM, Lorenzo E, Yamamura Y (2006) Detection of HERV-K(HML-2) viral RNA in plasma of HIV type 1-infected individuals. AIDS Res Hum Retroviruses 22: 979–984. 17067267 10.1089/aid.2006.22.979

[17] Contreras-Galindo R, Gonzalez M, modovar-Camacho S, Gonzalez-Ramirez S, Lorenzo E, et al (2006) A new Real-Time-RT-PCR for quantitation of human endogenous retroviruses type K (HERV-K) RNA load in plasma samples: increased HERV-K RNA titers in HIV-1 patients with HAART non-suppressive regimens. J Virol Methods 136: 51–57. 16678919 10.1016/j.jviromet.2006.03.029

[18] Contreras-Galindo R, modovar-Camacho S, Gonzalez-Ramirez S, Lorenzo E, Yamamura Y (2007) Comparative longitudinal studies of HERV-K and HIV-1 RNA titers in HIV-1-infected patients receiving successful versus unsuccessful highly active antiretroviral therapy. AIDS Res Hum Retroviruses 23: 1083–1086. 17919102 10.1089/aid.2007.0054

[19] Laderoute MP, Giulivi A, Larocque L, Bellfoy D, Hou Y, et al (2007) The replicative activity of human endogenous retrovirus K102 (HERV-K102) with HIV viremia. Aids 21: 2417–2424. 18025878 10.1097/QAD.0b013e3282f14d64

[20] Jones RB, John VM, Hunter DV, Martin E, Mujib S, et al (2012) Human endogenous retrovirus K(HML-2) Gag- and Env-specific T-cell responses are infrequently detected in HIV-1-infected subjects using standard peptide matrix-based screening. Clin Vaccine Immunol 19: 288–292. 22205657 10.1128/CVI.05583-11 PMC3272926

[21] Garrison KE, Jones RB, Meiklejohn DA, Anwar N, Ndhlovu LC, et al (2007) T Cell Responses to Human Endogenous Retroviruses in HIV-1 Infection. PLoS Pathog 3: e165. 17997601 10.1371/journal.ppat.0030165 PMC2065876

[22] SenGupta D, Tandon R, Vieira RG, Ndhlovu LC, Lown-Hecht R, et al (2011) Strong Human Endogenous Retrovirus-Specific T Cell Responses Are Associated with Control of HIV-1 in Chronic Infection. J Virol 85: 6977–6985. 21525339 10.1128/JVI.00179-11 PMC3126607

[23] Jones RB, Garrison KE, Mujib S, Mihajlovic V, Aidarus N, et al (2012) HERV-K-specific T cells eliminate diverse HIV-1/2 and SIV primary isolates. J Clin Invest 122: 4473–4489. 23143309 10.1172/JCI64560 PMC3533554

[24] Sacha JB, Kim IJ, Chen L, Ullah JH, Goodwin DA, et al (2012) Vaccination with cancer- and HIV infection-associated endogenous retrotransposable elements is safe and immunogenic. J Immunol 189: 1467–1479. 22745376 10.4049/jimmunol.1200079 PMC3401319

[25] Kammerer U, Germeyer A, Stengel S, Kapp M, Denner J (2011) Human endogenous retrovirus K (HERV-K) is expressed in villous and extravillous cytotrophoblast cells of the human placenta. J Reprod Immunol 91: 1–8. 21840605 10.1016/j.jri.2011.06.102

[26] Jager E, Karbach J, Gnjatic S, Neumann A, Bender A, et al (2006) Recombinant vaccinia/fowlpox NY-ESO-1 vaccines induce both humoral and cellular NY-ESO-1-specific immune responses in cancer patients. Proc Natl Acad Sci U S A 103: 14453–14458. 16984998 10.1073/pnas.0606512103 PMC1570182

[27] Gomez CE, Najera JL, Krupa M, Perdiguero B, Esteban M (2011) MVA and NYVAC as vaccines against emergent infectious diseases and cancer. Curr Gene Ther 11: 189–217. 21453284 10.2174/156652311795684731

[28] Staib C, Lowel M, Erfle V, Sutter G (2003) Improved host range selection for recombinant modified vaccinia virus Ankara. Biotechniques 34: 694–6. 12703290 10.2144/03344bm02

[29] Staib C, Drexler I, Sutter G (2004) Construction and isolation of recombinant MVA. Methods Mol Biol 269: 77–100. 15114009 10.1385/1-59259-789-0:077

[30] Hanke K, Kramer P, Seeher S, Beimforde N, Kurth R, et al (2009) Reconstitution of the ancestral glycoprotein of human endogenous retrovirus k and modulation of its functional activity by truncation of the cytoplasmic domain. J Virol 83: 12790–12800. 19812154 10.1128/JVI.01368-09 PMC2786854

[31] Chakrabarti S, Sisler JR, Moss B (1997) Compact, synthetic, vaccinia virus early/late promoter for protein expression. Biotechniques 23: 1094–1097. 9421642 10.2144/97236st07

[32] Kraus B, Monk B, Sliva K, Schnierle BS (2012) Expression of Human Endogenous Retrovirus-K Coincides with that of Micro-RNA-663 and -638 in Germ-cell Tumor Cells. Anticancer Res 32: 4797–4804. 23155245

[33] Lower R, Tonjes RR, Korbmacher C, Kurth R, Lower J (1995) Identification of a Rev-related protein by analysis of spliced transcripts of the human endogenous retroviruses HTDV/HERV-K. J Virol 69: 141–149. 7983704 10.1128/jvi.69.1.141-149.1995 PMC188557

[34] Mangeney M, Heidmann T (1998) Tumor cells expressing a retroviral envelope escape immune rejection in vivo. Proc Natl Acad Sci U S A 95: 14920–14925. 9843991 10.1073/pnas.95.25.14920 PMC24551

[35] Mangeney M, de Parseval N, Thomas G, Heidmann T (2001) The full-length envelope of an HERV-H human endogenous retrovirus has immunosuppressive properties. J Gen Virol 82: 2515–2518. 11562544 10.1099/0022-1317-82-10-2515

[36] Blaise S, Mangeney M, Heidmann T (2001) The envelope of Mason-Pfizer monkey virus has immunosuppressive properties. J Gen Virol 82: 1597–1600. 11413370 10.1099/0022-1317-82-7-1597

[37] Maurer-Gebhard M, Schmidt M, Azemar M, Altenschmidt U, Stocklin E, et al (1998) Systemic treatment with a recombinant erbB-2 receptor-specific tumor toxin efficiently reduces pulmonary metastases in mice injected with genetically modified carcinoma cells. Cancer Res 58: 2661–2666. 9635594

[38] Bannert N, Kurth R (2004) Retroelements and the human genome: new perspectives on an old relation. Proc Natl Acad Sci U S A 101 Suppl 214572–14579. 15310846 10.1073/pnas.0404838101 PMC521986

[39] Kleiman A, Senyuta N, Tryakin A, Sauter M, Karseladze A, et al (2004) HERV-K(HML-2) GAG/ENV antibodies as indicator for therapy effect in patients with germ cell tumors. Int J Cancer 110: 459–461. 15095315 10.1002/ijc.11649

[40] Humer J, Waltenberger A, Grassauer A, Kurz M, Valencak J, et al (2006) Identification of a melanoma marker derived from melanoma-associated endogenous retroviruses. Cancer Res 66: 1658–1663. 16452225 10.1158/0008-5472.CAN-05-2452

[41] van der Kuyl AC (2012) HIV infection and HERV expression: a review. Retrovirology 9: 6. 22248111 10.1186/1742-4690-9-6 PMC3311604

[42] Contreras-Galindo R, Kaplan MH, He S, Contreras-Galindo AC, Gonzalez-Hernandez MJ, et al.. (2013) HIV Infection Reveals Wide-Spread Expansion of Novel Centromeric Human Endogenous Retroviruses. Genome Res May 8. 10.1101/gr.144303.112 PMC3759726 23657884

[43] Gomez CE, Najera JL, Krupa M, Esteban M (2008) The poxvirus vectors MVA and NYVAC as gene delivery systems for vaccination against infectious diseases and cancer. Curr Gene Ther 8: 97–120. 18393831 10.2174/156652308784049363

[44] Ramirez JC, Gherardi MM, Esteban M (2000) Biology of attenuated modified vaccinia virus Ankara recombinant vector in mice: virus fate and activation of B- and T-cell immune responses in comparison with the Western Reserve strain and advantages as a vaccine. J Virol 74: 923–933. 10623755 10.1128/jvi.74.2.923-933.2000 PMC111613

[45] de Parseval N, Diop G, Blaise S, Helle F, Vasilescu A, et al (2005) Comprehensive search for intra- and inter-specific sequence polymorphisms among coding envelope genes of retroviral origin found in the human genome: genes and pseudogenes. BMC Genomics 6: 117. 16150157 10.1186/1471-2164-6-117 PMC1236922

[46] Reus K, Mayer J, Sauter M, Zischler H, Muller-Lantzsch N, et al (2001) HERV-K(OLD): ancestor sequences of the human endogenous retrovirus family HERV-K(HML-2). J Virol 75: 8917–8926. 11533155 10.1128/JVI.75.19.8917-8926.2001 PMC114460

[47] Sverdlov ED (2000) Retroviruses and primate evolution. Bioessays 22: 161–171. 10655035 10.1002/(SICI)1521-1878(200002)22:2<161::AID-BIES7>3.0.CO;2-X

